# Georgia State Dental Society

**Published:** 1893-09

**Authors:** J. M. Walker


### SOCIETY PROCEEDINGS.

### Georgia State Dental Society—Twenty-Fifth Annual Session, May, 1893.

Reported for the DENTAL REGISTER by Mrs. J. M. Walker.

The Georgia State Dental Society convened in twenty-fifth annual session in the parlors of the Kimball House, Atlanta, May 11, 1893.

Dr. N. N. Williams, of Valdosta, presided in the absence of the President, Dr. S. M. Roach, of Savannah.

The usual routine of addresses was marked by the absence of the customary presidential address due to the absence and ill health of the president.

The first subject of discussion was a proposed amendment of the by-laws in order to permit of more prompt action in the punishment of members found guilty of violation of the Code of Ethics. Two members of the society having been proven guilty of unprofessional advertising, and having failed to appear before the investigating committee after due notification, or to respond to communications from the committee, were dealt with according to the law of the society.

Dr. J. W. Daniels, Savannah, whose case had been under consideration at the preceding meeting, was, after due process, expelled from the society. Another member of the society, whose offense is of more recent date, was suspended for one year, thus giving him an opportunity to reinstate himself.

The Committee on Operative Dentistry presented a paper by Dr. J. C. Brewer, of Blackshear.

He dwelt, especially, upon the importance of properly caring for children's teeth, and the obligation of the dentist to enlighten parents on this subject; also the importance of gaining the full confidence of young patients and of never misleading them by false promises of painless operations.

He fills cavities in deciduous teeth with gutta-percha, capping with amalgam having a large percentage of tin, in the grinding surface of such teeth as have long to remain in position. When devitilization cannot be avoided he uses equal parts of carbolic acid and oil of cloves. He insisted upon the importance of training children in the proper use of the molars in mastication, and of furnishing them with food requiring mastication.

For permanent fillings in large cavities he has had good results from Daniel's Mastic, as a lining, forming an impervious coating which closes the mouth of the tubuli, and acts, to some extent, as a nonconductor. He uses cotton saturated with creasote and dipped in oxide of zinc for filling root canals. He does not consider pulp capping good practice, or "a safe road to travel," as he has been successful in only a small per cent. of cases. In all operations fair dealing should form the basis of action, keeping in view the elevation of the profession through the relief of suffering humanity.

Dr. B. F. Sims, of the same committee, read a paper entitled "Some of My Failings and the Causes."

Dr. Sims described his earlier methods of inserting gold fillings, pointing out the causes which led to leakage and failure, as in leaving too much of the thin enamel wall; operating from the labial surface; using gold in teeth of poor structure, etc. In his present method he carries the rubber-dam well down on the tooth with a separator, and cuts away all thin walls depending on under-cuts for retention of the filling. After polishing all the edges of the cavity with disks and strips he introduces noncohesive gold, from the palatal surface, in folds. After filling the cavity full of these folds he forces a plugger in between the folds as deep as possible and works in cohesive foil, relying upon wedging as well as cohesion. In this way he builds out the contour of the tooth and expects to save it. In teeth of poor structure he fills with cement, and gives instructions for the care of the mouth until the teeth become less sensitive and the tooth-structure improved in quality, possibly several years passing before he replaces the cement with permanent gold fillings. In conclusion he advised the young men of the profession not to ignore these failures, but to study them carefully, hoping to learn the lesson to be found in each. Successes will take care of themselves.

In the discussion of these papers Dr. O. H. McDonald took strong exceptions to Dr. Brewer's root-filling material. He uses either all gold, or gold at the apex followed by gutta-percha. He also prefers carbolic acid to coagulate the contents of the tubuli to the method of closing them with mastic. Also, he would not combine non-cohesive and cohesive gold in the same filling, as in his hands they break apart every time.

Dr. Sims replied that he found better retaining pits in the non-cohesive gold placed in the cavity than by drilling little holes in tooth substance.

The subjects of retaining pits, shaping cavities, modes of separating, etc., were discussed at some length by Drs. Catching, Tignor, Hall, Johnson, Jewett, Holland, Thompson, Barfield, Carpenter, Rosser, Browne, Holliday and others.

Dr. Catching relies on a properly shaped cavity for anchorage, starting his fillings with S. S. White's Crystal Mat Gold, building out with cohesive foil, using Bonwill's Electric, or the hand mallet.

Dr. Jewett wants one retaining point to hold his first piece of gold, getting further anchorage in under-cuts. By anchoring at the gingival margin you can build out and polish it down afterwards. He makes his own retaining point once and a half the diameter of the bur.

Dr. Frank Holland uses cohesive gold, thoroughly annealed, and starts with a pin-point drill for anchorage. Avoid weakening the walls by too deep under-cuts, or you cannot properly condense your gold, or will fracture enamel margins. Shape your cavity so that you can use everything you put into it, and knuckle your fillings so that enamel surfaces will not come in contact again.

Drs. Holland, W. G. Browne, Barfield, H. H. Johnson, Rosser and Jewett condemn severely the use of rubber in separating the teeth because of the extreme soreness it occasions, due to the elasticity of the rubber which is increased by occlusion. Cotton, tied with a ligature, laid between the teeth remains in position and separates the teeth through the absorption of water—hydraulic pressure—which is even and steady, holds the teeth firm in position, and occasions no soreness.

Drs. Thompson and Carpenter consider rubber for this purpose more cleanly than cotton, and if not too thick less objectionable than as stated by others. For immediate operations Dr. Holland uses Perry's Separators. Dr. Jewett prefers the separator to either rubber or cotton. If the blows of the mallet tend to drive the teeth apart another turn of the little wrench holds them firm and solid.

Drs. Carpenter and Tignor spoke at some length in favor of "Pyrozone," especially in the treatment of pyorrhea alveolaris, and as a general mouthwash, using the three per cent. solution for the latter purpose, and the ethereal solution for pyorrhea and for bleaching purposes.

Dr. Frank Holland does not concede any curative powers to peroxide of hydrogen. He considers that it acts simply as a cleanser, breaking up pus masses and washing them out.

Dr. W. G. Browne spoke of the beneficial action of nitrate of silver in arresting caries in the teeth of children, the very little ones for whom it is so difficult to properly prepare cavities for the retention of fillings.

Dr. R. Holliday uses it for this purpose in the shape of small pads of blotting paper soaked in a solution.

Dr. Catching uses it pulverized very finely.

Dr. Haynes applies it directly from the point of the stick in which it comes. He finds it very valuable in the worn-down teeth of old people.

In the discussion of Prosthetic Dentistry Dr. W. G. Browne described his method of making plates of pure, soft gold directly upon the plaster model without the use of metal dies, as given in the report of the Alabama Society, page 401. A patient wearing one of the plates was present and willing to have it examined by any one who so desired.

Dr. Thos. P. Hinman described the process of stamping aluminum crowns with the "Morrison Outfit." After the crowns are fitted and polished he thickens up the cusps with amalgam. These serviceable crowns can be stamped up in a few minutes, and for twenty cents enough aluminum plate can be bought to make "a hundred crowns." The great advantages offered are cheapness of material, ease and rapidity of manufacture, and the fact that they never tarnish.

The subject of "Crown-Work" was discussed by Drs. Rosser, Johnson, Thompson, Catching, W. H. Morgan, Crenshaw, Browne, D. D. Atkinson and Hinman.

Drs. Rosser, Johnson, Atkinson and Thompson prefer a band and cap crown to a seamless, stamped up crown, on the ground that you can see into an open band and be more certain of an accurate fit. The mandrels of a stamping outfit are uniform in shape, while teeth are infinite in variety, and in shaping a seamless crown with contour pliers the shape and articulation of the cusps are liable to be injured, also in filling the cusps there is danger of the solder flowing up the sides of the crown and spoiling the fit. They claim that if the cusps are filled in solidly and the band adjusted accurately before the two are joined there is more certainty of success. The difficulty of making the surface of the solder in the crown conform to the surface of the root was also spoken of.

To obviate the latter difficulty Dr. Thompson puts wax in the crown and places it on the root, the indentations in the wax showing where to grind the root. Red paint and a paste of rouge in glycerine are also used for this purpose. To prevent solder from flowing where it is not wanted Drs. Catching and Morgan use Spanish whiting and water, painting the surface where solder is not to flow.

Dr. Morgan spoke of the proper preparation of borax for soldering: heating it sufficiently to drive off the water of crystallization, or water absorbed from the atmosphere, which, forming steam puffs up the borax and causes the solder to crawl away from where you want it to stay. Dr. Morgan also spoke of the importance of making a crown long enough to cover all of the tooth from which the peridental membrane has been removed, thus affording protection against the ingress of foreign substances. The source of irritation being removed the teeth will assume healthy conditions, and loose teeth often become firm again in their sockets without any other treatment. He said: "I never hesitate now to put a crown on a root no matter how loose in the socket it may be."

Dr. Crenshaw spoke of the great convenience and utility of the seamless gold crowns now on the market. By getting them too large, if anything, and reducing with a reducing die (never attempting to stretch a crown that is too small) they can be made available in almost every possible case, and especially as dummies for masticating bridges. He gave in detail his method of removing a waxed up bridge from the mouth without getting the position of the teeth changed, or the piece bent, where there are several dummies.

Dr. Crenshaw fills a bridgework impression tray with quick-setting plaster and inverts it over the bridge as it is in position in the mouth, removing the bridge in the set plaster. He said: "Always place a pillow, or cushion of wax first of all from one cap crown to the other on the ridge, letting the teeth bear on the wax, and preventing the suspended crowns from sinking down and bearing on the ridge."

Dr. Thompson gave his method of articulating a crown. Having got a perfect fit for the band he places it on the root and fills it up high with wax, letting the patient bite down into it. This gives the exact articulation. Trim the wax and make a duplicate in Melotte's Metal, using a sand mold, wax up the cusps and trim it to fit accurately. Turn the open end of the band up and the cusps down and tack it to the top of the crown. With a sharp knife trim off all the gold except one point to hold it by. Catch it with pliers, holding the hollow end up, and you can put all the solder you choose in the cusps. The solder will always flow to the hottest point and it won't run up hill.

Dr. S. B. Cook, Chattanooga, Tenn., reported lack of success with cast aluminum plates, but that stamped plates are very satisfactory and pleasant to wear. He turns a rim on the edge of the plate in which he cuts grooves for rubber attachments.

Dr. Hinman uses gutta-percha to set Logan crowns.

Dr. D. D. Atkinson suggested the use of the Bonwill, the Ludwick and other crowns, saying that this society has been called "The Logan-Crown Association."

Dr. D. D. Atkinson, of the Committee on Education, read a paper on the question of "Unity of Action between the National and State Boards of Dental Examiners, and some Method of Inter-State Adjustment by which Competent Dentists may be enabled to remove from one State to another without undergoing an Examination."

Dental laws, like all others, are not retroactive and newly-created boards, being usually required to license all who are practicing at the time of the enactment of the law, regardless of fitness or qualifications. It is clear that the mere possession of a State license is no evidence that a man is competent to practice dentistry, and although legally qualified to practice within the border of his own State, it would not be proper to require other States to accept him on that ground. On the other hand, a State license based upon having passed an examination before a State Board, and backed by a diploma from a recognized college, would seem to afford evidence of qualifications entitling a man to practice wherever he may desire to reside.

This question was discussed by Drs. Wright, of South Carolina, Barfield, Rosser, Carpenter, Jewett, W. H. Morgan, of Nashville, Tenn., Catching and others, but no resolution was formed though all agreed that something ought to be done.

Dr. Catching read a paper on "The Persistent Effect of

Medicine Confined over the Dental Pulp." By this Dr. Catching refers to the common practice of placing on the floor of a cavity, having a layer of diseased dentine over a nearly exposed pulp, a medicament such as carbolic acid, creasote, one of the essential oils, or one of these mixed with oxide of zinc, the design being to guard against future pulp trouble.

Dr. Catching believes that the very thing sought to be guarded against by these means is in reality brought about by the procedure. The substances named are all penetrating, more or less volatile, and irritants when confined. The strata of softened, or diseased dentine, is soon penetrated and the pulp surface reached. The medicament must then either pass through the tooth substance or be taken up and carried off by the pulp. The latter is too much to expect and a struggle for life ensues in the very organ sought to be preserved. The period and mastery in this struggle is determined by the quantity, and the degree of irritating qualities of the medicament employed, by the vitality of the pulp and by the constitutionality of the patient, but as the usual result the medicament will prove the destroyer. Dr. Catching would treat a nearly exposed pulp by syringing the cavity thoroughly with as hot water as can be borne, dry out with absorbents and wash with bichloride of mercury in weak solution. Dry again with hot air, coat with varnish and fill.

Dr. W. G. Browne thought the method condemned by Dr. Catching the very best practice under the conditions and that the results were greatly exaggerated. The next best thing to do was to destroy the pulp and fill the root canals, which will have to be done eventually, and as well first as last, avoiding all intermediate trouble.

Dr. H. H. Johnson does not think that the medicaments named by the essayist, applied as described, ever cause the death of the pulp, that with or without them the pulp would die anyhow, the pulp being already sick and the disease of the dentine progressing to the pulp chamber.

Dr. W. H. Morgan says that when the layer of softened dentine is thoroughly dried, before the filling is inserted, there will be no further delay as there can be no disintegration without moisture. Dr. Morgan expressed surprise at what he termed "the inconsistency of rejecting the agents named, and yet employing corrosive sublimate—the most persisent and most active agent known to us, and one which expands when confined." Dr. Morgan advocates the use of carbolic acid, which causes the formation of a coagulum, preventing the continuance of decay and the decomposition of the semi-fluid contents of the tubuli. When there is persistent inflammation there is nothing better in the treatment of pulpless teeth than a combination of iodine and creasote, the former overcoming the odor of the latter when it is objected to.

Dr. S. W. Foster, Decatur, Ala., wished to ask Dr. Morgan what it is that causes the death of the pulp under a filling if decay always ceases with the insertion of the filling. His own idea is that the original disease continues to progress and kills the pulp.

Dr. Morgan attributes the death of the pulp under these circumstances to the pressure of the filling, or even to the irritation of the operation of filling, setting up chronic inflammation causing congestion of the pulp, the thickening of the membrane around the pulp arresting circulation and causing its death. You will never find a suppurating pulp without an outside opening. Strangulation cuts it off followed by decomposition, but there is no pus except from the outside. Suppuration is a physiological process and never occurs in dead bodies; the latter decompose but there is no pus.

Dr. H. H. Johnson laid before the society a preamble and resolutions looking to the appointment of a dental surgeon to the State Lunatic Asylum on the same footing as other assistant surgeons. This gave rise to a very interesting discussion of the influence of dental lesions with attendant reflex nervous action upon the brain as manifested in insanity. The subject was discussed at some length by Drs. Hinman, Rosser, Morgan, Johnson and others.

Dr. Rosser said that it was an admitted fact that the sufferings which dentistry only can relieve are a serious aggravation to the nervous condition of the inmates of lunatic asylums.

Dr. W. H. Morgan hoped the resolution would be adopted without a dissenting voice, that his attention had been drawn to the special condition of the teeth in connection with insanity, considered as a disease of the nervous system, and the mysteries of what is vaguely termed "reflex nervous action." He said that it is only occasionally that we stumble upon the location of the lesion which occasions it. The nerve of a tooth is a ganglion and is a frequent source of reflex nervous action; this is an admitted factor in the diseases of infancy while the deciduous teeth are making their appearance, and it is to our inability to control this action that is due the large proportion of infantile mortality during the second year. Diseases of the teeth may occasion this reflex nervous action at all ages, with the same general disturbances which are relieved by the same character of treatment. A large proportion of lunatics have defective teeth, and their brain trouble may be seriously aggravated by reflex nervous action from this source.

Dr. Morgan gave the history of several very interesting cases in which amelioration of brain troubles followed operations upon the teeth and, vice versa, when brain troubles resulted from too severe and prolonged operations upon the teeth, showing clearly the close relations between the two. He said: "Wherever nervous diseases are caused by severe irritation they come within the reach of medicine, and when caused by dental irritation the latter should be relieved by the dentist, as they come properly within his field of operations."

Dr. Johnson was made chairman of a committee appointed to bring this subject before the Board of Directors of the Georgia State Lunatic Asylum and secure their co-operation in this important matter.

The subject of how to secure papers for discussion was taken up and a resolution adopted requiring that all papers to be read at an annual meeting shall be submitted to a committee three months before the meeting, and fifty copies of all accepted papers be printed for distribution among the members that they may be prepared for an intelligent discussion.

The subject of collecting areas of dues was also discussed but no satisfactory solution of the vexed question was reached.

The election of officers resulted as follows: Dr. N. A. Williams, Valdosta, President; Dr. W. H. Hill, Washington, First Vice-President; Dr. C. V. Rosser, Atlanta, Second Vice-President; Dr. S. H. McKee, Americus, Recording Secretary; Dr. O. H. McDonald, Americus, Corresponding Secretary; Dr. H. A. Lawrence, Athens, Treasurer.

Drs. Atkinson, Bouton, Johnson, Coyle and Catching were elected as the next Board of Examiners.

Drs. Jewett, Hopps, Haynes, Simmons and Barfield were elected Executive Committee.

Tybee Island, in the mouth of the Savannah river, was the unanimous choice of the society for the next place of meeting, the selection of the time being left in the hands of the Executive Committee.

After the usual installation ceremonies the society adjourned *sine die.*

